# Vinpocetine Protects Against Cerebral Ischemia-Reperfusion Injury by Targeting Astrocytic Connexin43 via the PI3K/AKT Signaling Pathway

**DOI:** 10.3389/fnins.2020.00223

**Published:** 2020-04-02

**Authors:** Mingming Zhao, Shuai Hou, Liangshu Feng, Pingping Shen, Di Nan, Yunhai Zhang, Famin Wang, Di Ma, Jiachun Feng

**Affiliations:** ^1^Department of Neurology and Neuroscience Center, The First Hospital of Jilin University, Changchun, China; ^2^Suzhou Institute of Biomedical Engineering and Technology, Chinese Academy of Sciences, Suzhou, China; ^3^Jiangsu Key Laboratory of Medical Optics, Suzhou, China

**Keywords:** stroke, cerebral ischemia/reperfusion, oxygen-glucose deprivation/reoxygenation, vinpocetine, astrocyte, connexin 43, PI3K/AKT

## Abstract

Vinpocetine (Vinp) is known for its neuroprotective properties. However, the protective mechanism of Vinp against cerebral ischemia/reperfusion (I/R) injury should be further explored. This study was designed to investigate the neuroprotective effects of Vinp against oxygen-glucose deprivation/reoxygenation (OGD/R) injury *in vitro* and cerebral I/R injury *in vivo* and explore whether this mechanism would involve enhancement of astrocytic connexin 43 (Cx43) expression via the phosphatidylinositol 3-kinase/protein kinase B (PI3K/AKT) pathway. *In vitro*, we detected astrocytic viability and extracellular nitric oxide by an assay kit, intracellular reactive oxygen species by a DCFH-DA probe, inflammation and apoptosis-related protein expression by immunofluorescence staining, and the astrocytic apoptosis rate by flow cytometry. *In vivo*, we measured the cerebral infarction volume, superoxide dismutase activity, malondialdehyde content, and the expression of inflammation and apoptosis-related proteins. The results indicated that Vinp ameliorated the detrimental outcome of I/R injury. Vinp attenuated astrocytic injury induced by OGD/R and reduced cerebral infarction volume and cerebral edema in rats with cerebral I/R injury. Moreover, Vinp reduced oxidative stress, inflammation, and apoptosis induced by cerebral I/R injury in brain tissues. Meanwhile, Vinp increased p-Cx43 and p-AKT expression, and the p-Cx43/Cx43 and p-AKT/AKT ratio, which was decreased by cerebral I/R injury. Coadministration of PI3K inhibitors LY294002 and BKM120 blunted the effects of Vinp. This study suggests that Vinp protects against cerebral I/R injury via Cx43 phosphorylation by activating the PI3K/AKT pathway.

## Introduction

Ischemic stroke has high morbidity and mortality and seriously affects patient quality of life (Ribeiro et al., 2015). Timely recovery of blood and oxygen supply to the ischemic brain tissue is essential for ischemic penumbra survival. Thrombolytic therapy is the best treatment option for ischemic stroke (Sheth et al., 2015). However, reperfusion aggravates the damage and provokes dysfunction through a cascade of events such as calcium overload, excitotoxicity, oxidative stress, inflammatory responses, and apoptosis, which are collectively termed “ischemia-reperfusion injury” (I/R injury) (Dirnagl et al., 1999). Therefore, effectively blocking the cascade of cerebral I/R injury and exploring effective drugs for the treatment of ischemic stroke are very important.

Astrocytes are abundant in the central nervous system, and they play essential roles in maintaining brain function under physiologic conditions and in influencing neuronal survival under pathologic conditions, such as cerebral I/R injury and other brain insults (Garman, 2011; Falkowska et al., 2015; Verkhratsky et al., 2017). During ischemic stroke, astrocytes may be activated and produce and release reactive oxygen species (ROS), pro-inflammatory cytokines, and other factors that may negatively influence the survival of neurons in the penumbra (Swanson et al., 2004). Thus, preventing astrocytic inflammatory and apoptotic effects may be a promising strategy for neuroprotection in ischemic stroke (Cekanaviciute and Buckwalter, 2016; Choudhury and Ding, 2016; Liu and Chopp, 2016).

The PI3K/AKT signaling pathway regulates a wide range of cellular functions, including cellular differentiation, proliferation, inflammation, and apoptosis (Cantley, 2002). Studies have shown that phosphorylation of AKT (Ser473) reduces neuronal apoptosis caused by cerebral I/R injury (Fukunaga and Kawano, 2003; Zhao et al., 2006), and LY294002-mediated inhibition of the PI3K/AKT pathway blocked the cardioprotective effect of atorvastatin against I/R injury in cardiocytes by downregulating Connexin 43 (Cx43) (Bian et al., 2015). Moreover, activated AKT can phosphorylate the C-terminal Ser373 residue of Cx43 (Solan and Lampe, 2014). Since Cx43 is the most commonly expressed gap junction protein in astrocytes (Orellana et al., 2011), and increased Cx43 expression can reduce neuronal damage after cerebral I/R (Nakase et al., 2003), we speculate that Cx43 is involved in the PI3K/AKT pathway’s protective effects against cerebral I/R injury.

Vinpocetine (Vinp) is a semi-synthetic alkaloid derivative isolated from the leaves of *Phyllostachys pubescens*. Its anti-inflammatory and anti-platelet aggregation effects on improving cerebral blood flow, brain metabolism, and cognition have been confirmed by various studies (Zhang et al., 2018; Zhang et al., 2018). Vinp has been widely used in the treatment of stroke, cerebral arteriosclerosis, and chronic cerebral insufficiency, and it exhibits unique advantages in the treatment of dementia and epilepsy. A previous study showed that Vinp similarly decreased the inflammatory response by inhibiting NF-κB and TNF-α expression after cerebral I/R injury (Wang et al., 2014); however, its specific mechanism remains unknown. Cerebral I/R injury can activate both astrocytes and microglia, which may produce inflammatory cytokines and other toxic mediators (Kim et al., 2016; Duris et al., 2018). Microglial TLR4/MyD88/NF-κB has been shown to be one of the mechanisms by which Vinp protects against cerebral I/R injury (Wu et al., 2017). However, so far, no study has focused on whether Vinp’s protective effects against cerebral I/R injury is related to astrocytes. Hence, we hypothesized that Vinp may affect astrocytic Cx43 via the PI3K/AKT pathway and thereby provide neuroprotection.

In this study, we explored the neuroprotective roles of vinpocetine against oxygen-glucose deprivation/reoxygenation (OGD/R) injury *in vitro* and cerebral I/R injury *in vivo* and explore whether this mechanism would involve enhancement of astrocytic connexin 43 (Cx43) expression via the phosphatidylinositol 3-kinase/protein kinase B (PI3K/AKT) pathway.

## Materials and Methods

### Animal Care

The experiments adhered to the ethical standards of the Institutional Animal Care Committee and were approved by the Animals Ethics Committee of Jilin University. Male Wistar rats (250–280 g) and newborn rats were obtained from the Experimental Animal Center of Jilin University. Animals were maintained in a specific pathogen-free animal breeding room at 24°C under a 12 h day/night cycle with free access to water and food. All possible measures were taken to avoid animals suffering at each stage of the experiment.

### Primary Rat Astrocytic Culture

Astrocytes were obtained from the cerebral cortex of newborn rats as previously described (Schildge et al., 2013). Newborn Wistar rats were decapitated, and the cerebral cortices were isolated in cold Dulbecco’s Modified Eagle Media: Nutrient Mixture F-12 (DMEM/F12) medium. Then, the meninges were carefully removed, and the tissues were treated with 0.125% trypsin solution for 15 min at 37°C. DMEM/F12 containing 10% fetal bovine serum (FBS) was added, and the mixture was centrifuged at 1300 rpm for 5 min. The sediment was resuspended with DMEM/F12 containing 10% FBS. At a concentration of 10^5^/ml, cells were planted onto 75 cm^2^ flasks in 15 ml DMEM/F12 containing 10% FBS and 1% penicillin/streptomycin and placed in an incubator (Thermo Scientific, Waltham, MA, United States) at 37°C with 95% air and 5% CO_2_. After 24 h, the medium was changed in the flasks, and then half of the medium was changed every 3 days. After approximately 12 days, the astrocytic cultures reached confluency. Oligodendrocytes and microglia were deprived from astrocytic cultures by shaking on an orbital shaker for 6 h at 37°C (Schildge et al., 2013). The astrocytic cultures were treated with 0.25% trypsin solution for 3 min at 37°C. Then, the cells were harvested, and they were adjusted to a density of 2 × 10^5^ cells/ml and planted on flasks. The third generation of primary cultured astrocytes were used in our study. The purity of astrocytes was higher than 95%, as confirmed by immunofluorescence staining with a specific marker, the glial fibrillary acidic protein (GFAP) (ab7260, Abcam, United States). A representative result is shown in Supplementary Figure S1A.

### Oxygen-Glucose Deprivation/Reoxygenation (OGD/R) *in vitro* Model

As described previously (Ferrer-Acosta et al., 2017), oxygen-glucose deprivation/reoxygenation (OGD/R) is a classic *in vitro* model of I/R injury. Briefly, astrocytes were washed three times with glucose-free DMEM and cultured in the same medium in a hypoxia chamber with a mixture of 95% N_2_ and 5% CO_2_ for 12 h. Then, the astrocytes were cultured in normal DMEM medium and re-oxygenated under normoxic conditions (95% air, 5% CO_2_) for 6 h.

The astrocytic cultures were divided into five groups: (1) a control group, stimulated with DMSO; (2) an OGD/R group, stimulated with DMSO during OGD/R injury; (3) an OGD/R + Vinp group, stimulated with Vinp (30 μM) (Gedeon Richter Pharmaceutical Co., Ltd., Budapest, Hungary) during OGD/R injury; and (4) an OGD/R + Vinp + LY group, stimulated with LY294002 (20 μM) (ab120243, Abcam, Cambridge, MA, United States) and Vinp during OGD/R injury; (5) OGD/R + Vinp + BKM group, stimulated with BKM120 (2 μM) (S2247, Selleck, Houston, TX, United States). LY and Vinp were dissolved in DMSO at a final concentration of 100 mM (Hong et al., 2013; Takac et al., 2013; Nivison-Smith et al., 2015), and BKM was dissolved in DMSO at a final concentration of 10 mM. As described above, all groups were stimulated with the same volume of DMSO, and for the control group 0.33% DMSO proved to have no obvious toxicity on astrocytes (Supplementary Figure S1B).

### Cell Viability and Cytotoxicity Assay

Commercial cell counting Kit-8 (CCK-8) (Do-jindo, Kumamoto, Japan) was used to detect cell viability (Ishiyama et al., 1997). Primary astrocytes cultured to the third generation were seeded in 96-well plates at a density of 10^4^/well. The 96-well plates were placed in a cell culture incubator for 24 h before being subjected to OGD/R. Thereafter, 10 μL CCK-8 reagent was added to each well. The 96-well plates were then placed in the cell culture incubator for 2 h, and the absorbance at 450 nm was measured by a microplate reader (Multiskan, Thermo Scientific, Waltham, MA, United States).

Cytotoxicity was determined by measuring the lactate dehydrogenase (LDH) of the cell culture supernatant using the Cytotoxicity Detection Kit (C0016, Beyotime, Shanghai, China) according to the manufacturer’s instructions (Lobner, 2000). Briefly, the sample maximum enzyme activity control wells were set according to the instructions. Astrocytic supernatants from each group were centrifuged. In each well of the 96-well plate was added 120 μL supernatant and 60 μL reagent. Then, the 96-well plate was incubated at room temperature for 30 min in the dark, and the absorbance at 490 nm was measured by a microplate reader. Experiments were repeated five times, and each experiment contained five duplicate wells for each astrocyte group.

### Detection of Intracellular ROS and Extracellular NO

The ROS Assay kit (S0033, Beyotime) was used to detect ROS in astrocytes (Eruslanov and Kusmartsev, 2010). Briefly, astrocytes were seeded at a density of 104 cells/well in 96-well plates. After exposure to OGD/R injury, 10 μM of DCFH-DA in serum-free DMEM medium was added to each well. After incubation for 30 min in the cell culture incubator, each well was washed three times with serum-free DMEM and examined by a microplate reader using excitation/emission wavelengths of 488/525 nm.

Astrocytic Nitric oxide (NO) release was detected using the NO Assay Kit (S0021, Beyotime) (Weissman and Gross, 2001). Astrocytes were seeded in 96-well plates. A total of 50 μL/well of Griess Reagent I and 50 μL/well of Griess Reagent II were added into each well immediately after the astrocytes were exposed to OGD/R injury. The standard curve was constructed according to the instructions. The absorbance at 540 nm was measured by a microplate reader.

### Astrocytic Immunofluorescence Analysis

Astrocytes were fixed with 4% paraformaldehyde at room temperature for 30 min and washed three times with PBS. After permeabilization with 0.2% Triton X-100 for 10 min and blocking with 10% goat serum in PBS for 1 h, the cells were incubated with rabbit anti-IL-1β (ab9722, Abcam; 1: 100), anti-TNF-α (ab66579, Abcam; 1: 100), anti-Bcl-2 (ab194583, Abcam; 1: 50), and anti-caspase-3 antibodies (ab13847, Abcam; 1:50) overnight at 4°C, followed by incubation with goat anti-rabbit IgG Fc (Alexa Fluor 647, ab150091, Abcam; 1:200) for 2 h at 25°C. The cells were then incubated with DAPI for 5 min and examined under a fluorescence microscope (OLYMPUS BX51, Tokyo, Japan).

### Astrocytic Apoptosis Assay

Apoptosis was assessed by flow cytometry using an FITC Annexin V Apoptosis Detection Kit I (556547, Becton Dickinson, Franklin Lakes, NJ, United States) according to the manufacturer’s instructions (Frey, 1997). Briefly, cells were rinsed with ice-cold PBS and then resuspended in 100 μL binding buffer (10^5^ cells). A total of 5 μL Annexin V and 5 μL PI were added to each sample, and they were incubated for 15 min at 25°C in the dark. Then, 400 μL binding buffer was added to each tube and cells were immediately analyzed using a FACSC-LSR (Becton Dickinson) and evaluated with the Flow Jo 7.6 software.

### Middle Cerebral Artery Occlusion (MCAO) Model and Animal Grouping

The MCAO model, a classic *in vivo* model of I/R injury, was prepared as previously described (Longa et al., 1989). Briefly, Wistar rats were anesthetized with chloral hydrate (350 mg/kg, i.p.). Then, a midline incision in the neck was made to expose the left external and internal arteries (ECA and ICA). The ECA was cut between two ligations, and a 0.26 mm silicone-tipped filament (2636, Xinlong lnc., Beijing, China) was inserted into the ICA via the ECA at approximately 20 mm until a resistance was felt, which ensured the occlusion of the MCA. Then, the suture was tightened around the ECA stump and the incision was closed. After surgery, rats remained in the cage for 2 h. Then, the animals were anesthetized again, and the filament was removed. During surgery, the rats’ body temperature was maintained at a normal level by heating pads. After awakening, the rats were maintained in cages with free access to food and water for 12 h.

A total of 64 male Wistar rats were randomly divided into four groups: (1) a sham group: the rats were injected with 0.9% normal saline and were not subjected to MCAO; (2) an I/R group: the rats were injected with 0.9% normal saline and subjected to MCAO; (3) a Vinp + I/R group: the rats were injected with Vinp (10 mg/kg) and subjected to MCAO; and (4) a Vinp + I/R + LY group: the rats were initially injected with LY294002 (0.3 mg/kg) and then with Vinp 15 min later and subjected to MCAO. All injections were administered intraperitoneally 30 min prior to MCAO.

### Neurological Evaluation

Neurological evaluation was performed after 2 h of ischemia and 12 h of perfusion by a researcher blinded to the experimental groups. Evaluation was performed using a modified form (Longa et al., 1989) as follows: (0) no deficits; (1) difficulty to fully extend the contralateral forelimb; (2) inability to extend the contralateral forelimb; (3) mild circling to the contralateral side; (4) severe circling; and (5) falling to the contralateral side. Finally, the rats were anesthetized and decapitated for the brain water content assay, TTC staining, western blot, immunofluorescence, SOD activity, and MDA content analyses.

### TTC Staining

2,3,5-triphenyltetrazolium chloride (TTC, Sigma, St. Louis, MI, United States) staining was used to visualize the ischemic infarction (Bederson et al., 1986). After decapitation, the brains were sliced into 2 mm sections, and each slice was incubated in a 2% solution of TTC at room temperature for 20 min and fixed in 4% paraformaldehyde. The brain sections were photographed using a high-resolution digital camera (Olympus). The infarct size was measured using the Image J software (NIH Image, National Institutes of Health, Bethesda, MD, United States). The percentage of the infarction size was calculated as described previously (Jackman et al., 2011).

### Brain Water Content Assay

The classic wet-dry method was used to measure brain water content (Agrawal et al., 1968). Immediately after the rats were sacrificed, the brains were taken and weighed to obtain the wet weight. The samples were then dried in an oven at 100°C for 48 h. They were then weighed again to obtain the dry weight. Water content = (wet weight−dry weight)/wet weight × 100%.

### Measurement of SOD Activity and MDA Content

Commercially available detection kits (Nanjing Jiancheng Bioengineering Institute, Nanjing, China) were used to detect SOD activity and MDA content according to the manufacturer’s instructions as previously described (Hou et al., 2016). Briefly, SOD activity was assessed using the xanthine oxidase method, and MDA content was measured with the thiobarbituric acid method. The samples were analyzed with a spectrophotometer (BioRad, San Diego, CA, United States).

### Immunofluorescent Analysis of Brain Sections

After decapitation, the brains were harvested immediately, immersed into pre-chilled isopentane (Beijing Chemical Factory, Beijing, China), and placed inside a −80°C refrigerator for 10 min for snap-freezing. Then, the brains were embedded in optimum cutting temperature compound (Sakura Finetek Inc., Torance, CA, United States) and stored in the −80°C refrigerator. Subsequently, 10-μM section of the brain were obtained using a cryomicrotome (Leica, Nussloch, Germany). The sections were fixed with 4% paraformaldehyde at room temperature for 15 min and washed three times with PBS. After permeabilization with 1% Triton X-100 for 10 min and subsequent blocking with 10% goat serum in PBS for 1 h, the sections were incubated with mouse anti-GFAP (ab10062, Abcam, 1:500) and rabbit anti-TNF-α (ab66579, Abcam; 1: 200) overnight at 4°C, followed by incubation with Alexa Fluor 647-conjugated goat anti-mouse IgG (ab150115, Abcam; 1:200) and Alexa Fluor488-conjugated goat anti-rabbit IgG (ab150077, Abcam; 1:200) for 1 h at 25°C. Experimental negative control was a section without any primary antibody treatment. The slices were then incubated with DAPI for 5 min and examined under a confocal microscope (Leica TCS SP5, Nussloch, Germany).

### Western Blot Analysis

The western blot analysis was conducted as previously described (Hou et al., 2016). The cortex in the same set of rats or the cultured astrocytes was crumbled and homogenized with ice-cold lysis buffer (RIPA: NaVO3: PMSF: NaF = 92:5:2:1). Proteins were extracted from the cerebral cortex tissue, and the protein concentrations were assayed. Each sample (50 μg) was loaded on a 12% sodium dodecyl sulfate polyacrylamide gel electrophoresis apparatus and electrophoresis was carried out until the bromophenol blue dye reached the bottom of the gel. Then, the proteins were electro-transferred to polyvinylidene fluoride membranes, and the membranes were placed in 5% skim milk powder dissolved in TBS with 0.1% Tween-20 for 1 h. The membranes were incubated with anti-Cx43 (ab11370, Abcam; 1:1000), anti-p-Cx43 (PA5-37584, Thermo Fisher Scientific; 1:1000), anti-AKT (4691, Cell Signaling Technology, Danvers, MA, United States; 1:1000), anti-p-AKT (13038, Cell Signaling Technology; 1:1000), anti-IL-1β (ab9722, Abcam; 1: 1000), anti-TNF-α (ab66579, Abcam; 1: 1000), anti-IL-10 (ab9969, Abcam; 1:2000), anti-Bcl-2 (ab194583, Abcam; 1:500), anti-caspase-3 (ab13847, Abcam; 1:500), and anti-β-actin (ab13847, Abcam; 1:2000) antibodies diluted in 5% skim milk powder dissolved in TBST overnight at 4°C. The membranes were then washed with PBST and incubated with a horseradish peroxidase-conjugated secondary antibody for 1 h. The protein bands were quantified with the Quantitation One software (Bio-Rad Laboratories, Hercules, CA, United States).

### Statistical Analysis

All data are presented as the mean ± standard error of the mean (SEM) from at least three independent experiments using Graphpad Prism 6 (Inc., San Diego, CA, United States). Analysis was carried out by one-way analysis of variance (ANOVA) followed by Tukey’s *post hoc* tests. ^∗^*P* < 0.05, ^∗∗^*P* < 0.01 or ^∗∗∗^*P* < 0.001 denoted the significance thresholds.

## Results

### Vinp Increased Astrocytic Viability and Attenuated Astrocytic Injury Induced by OGD/R

**FIGURE 1:**
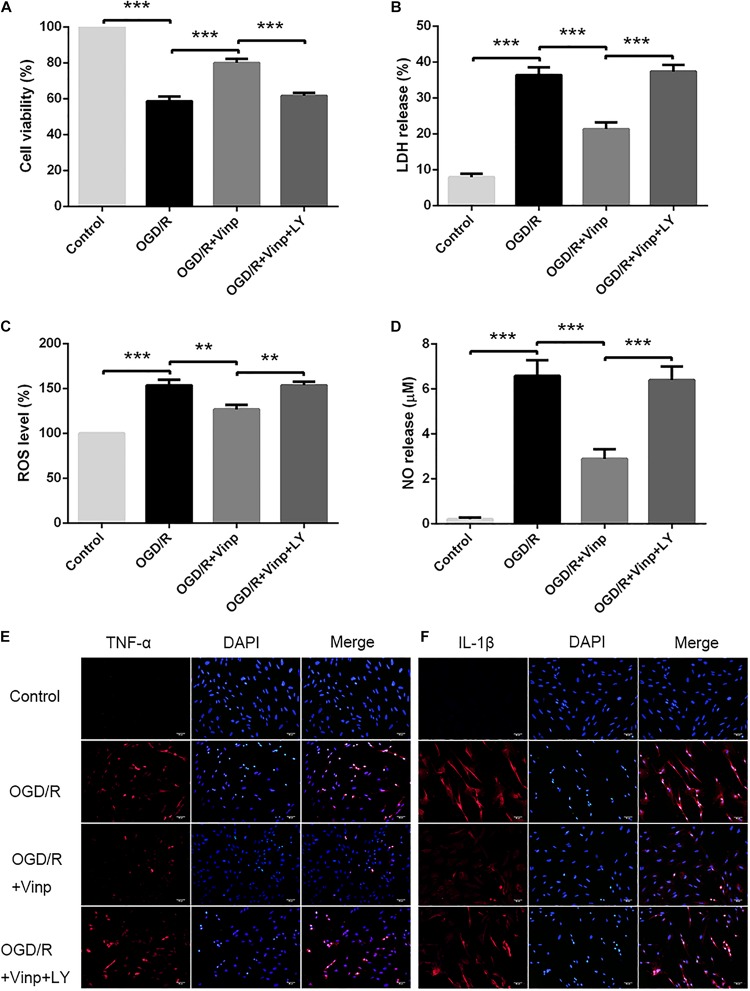
Effects of Vinp on astrocytic viability, cytotoxicity, oxidative stress, and inflammation after OGD/R. **(A)** Astrocytic viability calculated as a percentage relative to the control group (*n* = 5 in each group). **(B)** LDH release calculated as a percentage relative to maximum enzyme activity control well (*n* = 5 in each group). **(C)** ROS levels calculated as a percentage relative to the control group (*n* = 5 in each group). **(D)** NO released into the supernatant evaluated by NO assay kit (*n* = 5 in each group). **(E)** Immunostaining showed the expression of TNF-α in astrocytes after OGD/R. **(F)** Immunostaining showed the expression of IL-1β in astrocytes after OGD/R, and the nuclei were counterstained with DAPI (*n* = 3 in each group). Scale bars = 50 μm. Data are shown as the mean ± SEM. **P < 0.01, ***P < 0.001 using one-way ANOVA followed by Tukey’s *post hoc* tests. Vinp: Vinpocetine; LY: LY294002; OGD/R: oxygen-glucose deprivation/reoxygenation.

To investigate the neuroprotective effects of Vinp in primary cultured astrocytes *in vitro*, we assessed cell viability in each group via the CCK-8 assay (Figure 1A). The results showed that approximately half of the astrocytes survived in OGD/R group compared with the control group (56.86 ± 2.62%, *P* < 0.001). Compared with OGD/R group, viability was significantly improved in astrocytes treated with OGD/R + Vinp (78.94 ± 2.78%, *P* < 0.001). However, this elevation was reversed in the OGD/R + Vinp + LY group compared with the OGD/R + Vinp group (61.77 ± 2.09%, *P* < 0.001). We also measured astrocytic injury by testing the amount of LDH released into the supernatant. This test showed that OGD/R injury significantly increased the release of LDH compared with that in the control group (35.77 ± 2.60% vs. 8.62 ± 0.75%, *P* < 0.001). Treatment with OGD/R + Vinp remarkably decreased the release of LDH compared with that in the OGD/R group (20.14 ± 1.99%, *P* < 0.001). In comparison with OGD/R + Vinp group, coadministration of LY294002, a PI3K inhibitor, resulted in an apparent increased release of LDH (38.63 ± 1.81%, *P* < 0.001). These findings indicated that Vinp could promote cell survival and reduce cell damage in astrocytes subjected to OGD/R, and the inhibition of the PI3K/AKT pathway could abolish the protection of Vinp.

### Vinp Attenuated Oxidative Stress in Astrocytes Induced by OGD/R Injury

Increased ROS production is considered an initial step in OGD/R injury (Dirnagl et al., 1999). To examine the effect of Vinp on OGD/R injury-induced oxidative stress in astrocytes, we detected intracellular ROS and NO released into the extracellular supernatant (Figures 1C,D). The results showed that intracellular ROS and extracellular NO was significantly increased after OGD/R injury compared with the control group (*P* < 0.001), but this elevation was reversed in the OGD/R + Vinp group compared with the OGD/R group (*P* < 0.01). Compared with the OGD/R + Vinp group, intracellular ROS and extracellular NO in the OGD/R + Vinp + LY group was markedly increased (*P* < 0.01). The data revealed that Vinp decreased oxidative stress in astrocytes induced by OGD/R injury, which was attenuated by a PI3K/AKT pathway inhibitor (LY294002).

### Vinp Alleviated Inflammatory Cytokine Expression in Astrocytes After OGD/R Injury

As previously mentioned, the large amounts of ROS generated during OGD/R can induce inflammation by activating astrocytes (Chan, 2001; Duris et al., 2018). To investigate the anti-inflammatory effects of Vinp, we used immunofluorescence staining to observe the expression of TNF-α and IL-1β (Figures 1E,F). OGD/R injury resulted in significant increase in astrocytic TNF-α and IL-1β expression compared with control group, and this increase was blocked by treatment with Vinp during OGD/R injury. Moreover, LY reversed the effect of Vinp on astrocytes subjected to OGD/R, significantly increasing TNF-α and IL-1β expression. The results indicated that the PI3K/AKT pathway is involved in the anti-inflammatory effects of Vinp against OGD/R injury.

### Vinp Altered Apoptosis-Related Protein Expression and Reduced the Apoptotic Rate in Astrocytes After OGD/R Injury

**FIGURE 2:**
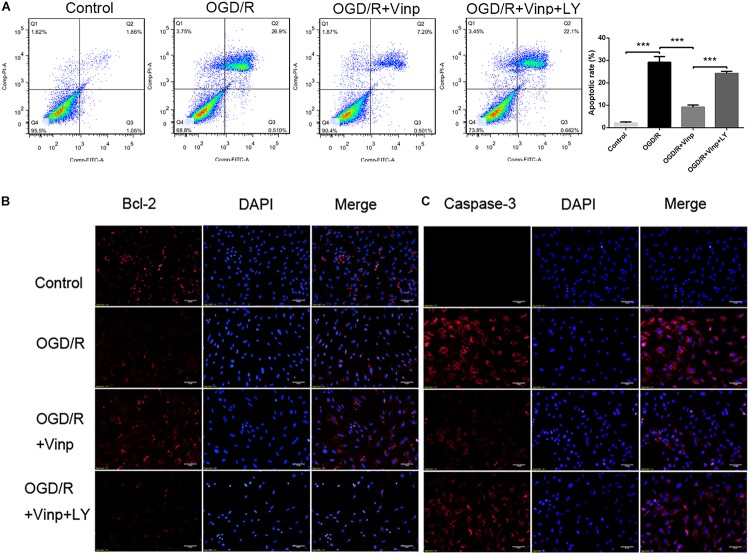
Effects of Vinp on astrocytic apoptosis after OGD/R. **(A)** Immunostaining showed the expression of Bcl-2 and caspase-3 in astrocytes after OGD/R. **(B)** Immunostaining showed the expression of Bcl-2 and caspase-3 in astrocytes after OGD/R. The nuclei were counterstained with DAPI. Scale bars = 50 μm. **(C)** Representative flow cytometry images of apoptosis observed with Annexin V FITC/PI staining. **(D)** Apoptosis analysis calculated as a percentage relative to total cells. Data are shown as the mean ± SEM (*n* = 3 in each group). ***P < 0.001 using one-way ANOVA followed by Tukey’s *post hoc* tests. Vinp: Vinpocetine; LY: LY294002; OGD/R: oxygen-glucose deprivation/reoxygenation.

As a cascade event, increased ROS may induce excessive inflammatory responses which could activate pro-apoptotic pathways (Duris et al., 2018), and therefore we evaluated the anti-apoptotic effects of Vinp. Annexin V FITC/PI staining and flow cytometry were used to detect the astrocytic apoptotic rate (Figure 2A), and immunofluorescence staining was processed to observe the expression of caspase-3 and Bcl-2 (Figures 2B,C). We found that OGD/R injury resulted in a significant increase in astrocytic caspase-3 expression, a significant reduction in Bcl-2 expression, and an increase in apoptotic rate compared to the control group (*P* < 0.001). Compared with the OGD/R group, caspase-3 expression and astrocytic apoptotic rate effectively decreased while Bcl-2 expression increased in the OGD/R + Vinp group (*P* < 0.001). Furthermore, results showed LY reversed the effect of Vinp on OGD/R-treated astrocytes, significantly increasing astrocytic caspase-3 expression, reducing Bcl-2 expression, and increasing apoptotic rate (*P* < 0.001). These results indicated that the PI3K/AKT pathway is involved in the anti-apoptotic effects of Vinp against OGD/R injury.

### Vinp Decreased Brain Water Content and Infarction Size in Rats After Cerebral I/R Injury

**FIGURE 3:**
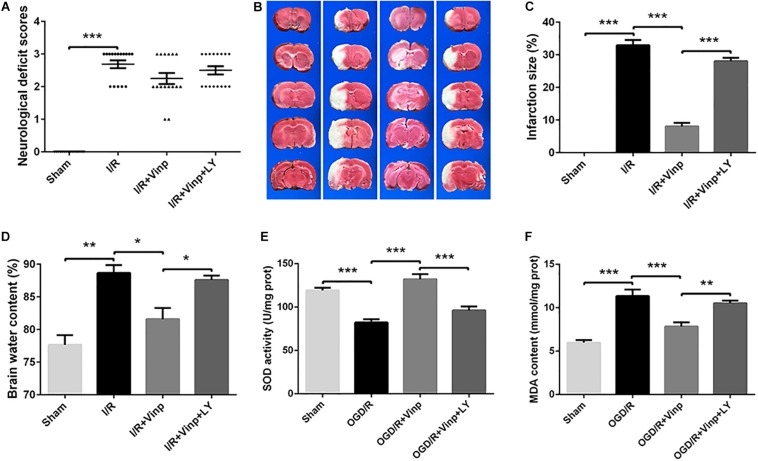
Effects of Vinp on infarction size, neurological deficits, brain water content, and oxidative stress in rats following MCAO. **(A)** Neurological deficit analysis (*n* = 16 in each group). **(B)** Brain water content analysis (*n* = 3 in each group). **(C)** Representative images of cerebral infarction after ischemia for 2 h and reperfusion for 12 h in rat brains by TTC staining. **(D)** Analysis of infarct size calculated as a percent relative to total cerebral volume (*n* = 3 in each group). **(E)** Analysis of SOD activity (*n* = 5 in each group). **(F)** Analysis of MDA content (*n* = 5 in each group). *P < 0.05, **P < 0.01, ***P < 0.001 using one-way ANOVA followed by Tukey’s *post hoc* tests. Vinp: Vinpocetine; LY: LY294002; I/R: ischemia/reperfusion; MCAO: middle cerebral artery occlusion; TTC: 2,3,5-triphenyltetrazolium chloride.

Next, we prepared the classic MCAO models to evaluate the early effects of Vinp against cerebral I/R injury *in vivo*. As previously described, neurological deficit scores, brain water content, and infarction size were measured to evaluate the effects of Vinp against cerebral I/R injury after the rats were subjected to ischemia for 2 h and reperfusion for 12 h. In the I/R group, there were obvious symptoms of neurological deficits including deviation to the right, circling, and inability to fully extend the right upper limb. Treatment with Vinp did not significantly reduce the neurological deficit scores (Figure 3A, *P* = 0.18). The cerebral infarct size after MCAO is shown in Figures 3B,C. In the I/R group, significant cerebral infarction was observed compared with the sham group (32.92 ± 1.63%, *P* < 0.001), and this phenotypic alteration was mostly abrogated in the I/R + Vinp group compared with the I/R group (8.06 ± 1.10%, *P* < 0.001). Compared to the I/R + Vinp group, infarct size in the I/R + Vinp + LY group was significantly enlarged (28.04 ± 1.05%, *P* < 0.001). The results of the water content of brain tissues were consistent with the trend observed for infarction size (Figure 3D). The results revealed that Vinp decreased the infarction size and brain edema, while inhibition of PI3K/AKT reversed the protection of Vinp.

### Vinp Attenuated Oxidative Stress in the Rat Cerebral Cortex After Cerebral I/R Injury

We further examined oxidative stress in ischemic cerebral cortices as the *in vitro* study, which is considered the initial step of cerebral I/R injury. SOD activity is an important antioxidant enzyme, and MDA content reflects oxidative damage (Chan, 2001), therefore, we examined SOD activity and MDA content (Figures 3E,F). Compared to the sham group, SOD activity significantly decreased, while MDA content increased in the I/R group (*P* < 0.001). Treatment with Vinp effectively increased SOD activity and decreased MDA content compared with the I/R group (*P* < 0.001), whereas LY reversed the effects of Vinp by decreasing SOD activity and increasing MDA content compared with the I/R + Vinp group (*P* < 0.01). The above findings suggested that Vinp attenuated oxidative stress induced by cerebral I/R injury, which is related to the PI3K/AKT pathway.

### Vinp Reduced Inflammation and Apoptosis in the Rat Cerebral Cortex After Cerebral I/R Injury

**FIGURE 4:**
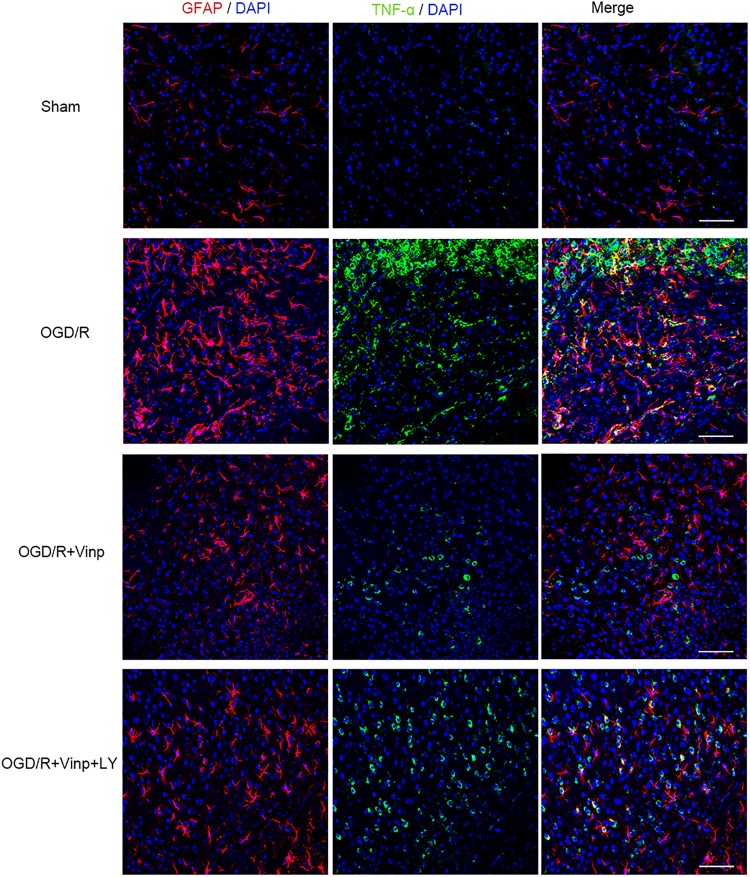
Effects of Vinp on reactive astrocytes and TNF-α in the rat cerebral cortex after I/R injury. Brain slices were analyzed with double immunostaining using GFAP (red, a reactive astrocytic marker) and TNF-α (green, a pro-inflammatory cytokine). The nuclei were stained with DAPI (*n* = 3 in each group). Scale bars = 50 μm. Vinp: Vinpocetine; LY: LY294002; I/R: ischemia/reperfusion;

**FIGURE 5:**
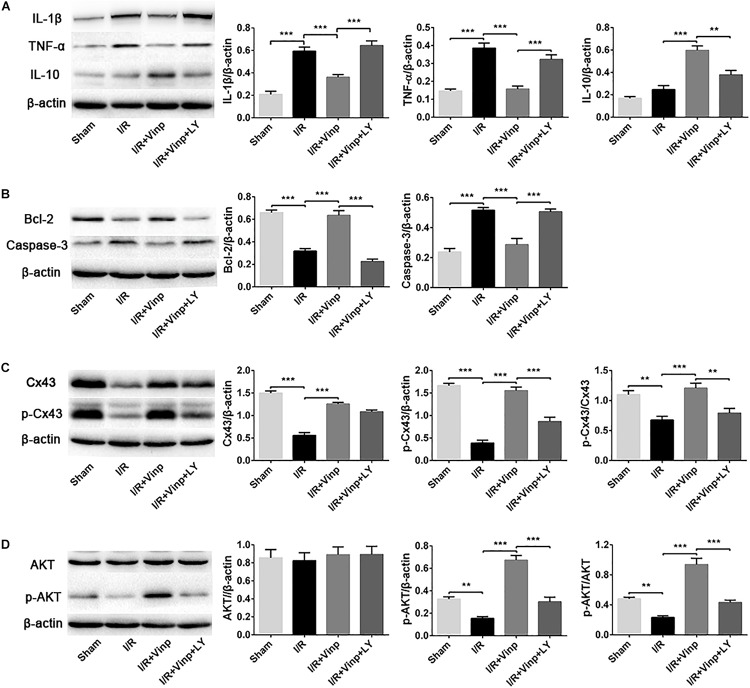
Effects of Vinp on inflammation and apoptosis-related proteins, and the expression of Cx43, p-Cx43, AKT, and p-AKT in the rat cerebral cortex after I/R injury. **(A)** Representative western blot images and quantification of proteins related to inflammation. **(B)** Representative western blot images and quantification of proteins related to apoptosis. **(C)** Representative western blot images of Cx43 and p-Cx43, and quantification of Cx43, p-Cx43, and p-Cx43/Cx43. **(D)** Representative western blot images of AKT and p-AKT, and quantification of AKT, p-AKT and p-AKT/AKT. Data are shown as the mean ± SEM (*n* = 5 in each group). **P < 0.01, ***P < 0.001 using one-way ANOVA followed by Tukey’s *post hoc* tests. Vinp: Vinpocetine; LY: LY294002; I/R: ischemia/reperfusion.

To validate if the response cascade was caused by cerebral I/R injury, we examined the inflammation and apoptosis *in vivo*. First, we performed immunofluorescent analysis to observe the reactive astrocytes and inflammatory cytokine by double immunostaining the brain cryosections with anti-GFAP and anti-TNF-α antibodies (Figure 4). Cerebral I/R injury resulted in a significant increase in the expression of GFAP and TNF-α, and the co-localization of GFAP and TNF-α was compared with that in the sham group. However, this increase was blocked by Vinp treatment. Furthermore, LY reversed the effect of Vinp in reactive astrocytes, significantly increasing the expression of GFAP and TNF-α, and consequently, the co-localization of GFAP and TNF-α. The results showed that Vinp treatment significantly decreased cerebral I/R injury-induced inflammation by reducing astrocyte activation. Then, western blot analysis was used to detect the expression of inflammation-associated proteins, IL-1β, TNF-α, and IL-10, and apoptosis-related proteins, caspase-3 and Bcl-2 (Figures 5A,B). IL-1β, TNF-α, and caspase-3 expression significantly increased while Bcl-2 expression significantly decreased in the I/R group compared with the sham group (*P* < 0.001). IL-1β, TNF-α, and caspase-3 expression decreased while IL-10 and Bcl-2 expression increased in the I/R + Vinp group compared to the I/R group (*P* < 0.001). Conversely, LY blocked the above effects of Vinp, significantly increasing IL-1β, TNF-α, and caspase-3 expression while decreasing IL-10 and Bcl-2 expression (*P* < 0.01). Overall, these results indicated that the PI3K/AKT pathway is involved in the anti-inflammatory and anti-apoptotic effects of Vinp against cerebral I/R injury.

### Vinp Activated p-Cx43 via the PI3K/AKT Pathway in the Rat Cerebral Cortex After Cerebral I/R Injury

In order to explore whether the above protective effects of Vinp are exerted by targeting Cx43 via the PI3K/AKT pathway, we examined the expression of Cx43, p-Cx43, AKT, and p-AKT. Compared to the sham group, I/R injury significantly repressed the expression of Cx43 and p-Cx43 and the p-Cx43/Cx43 ratio (Figure 5C, *P* < 0.01). Interestingly, Vinp could increase Cx43 and p-Cx43 expression and the p-Cx43/Cx43 ratio compared with the I/R group, indicating the activation of the Cx43 (*P* < 0.001). However, significant reduction of p-Cx43 expression and the p-Cx43/Cx43 ratio was observed in the I/R + Vinp + LY group compared with I/R + Vinp group (*P* < 0.01). The expression of p-AKT and the ratio of p-AKT/AKT were similar to those of p-Cx43 and p-Cx43/Cx43, while the AKT levels did not significantly differ among the groups (Figure 5D).

### Vinp Activated p-Cx43 via the PI3K/AKT Pathway in Astrocytes After OGD/R Injury

**FIGURE 6:**
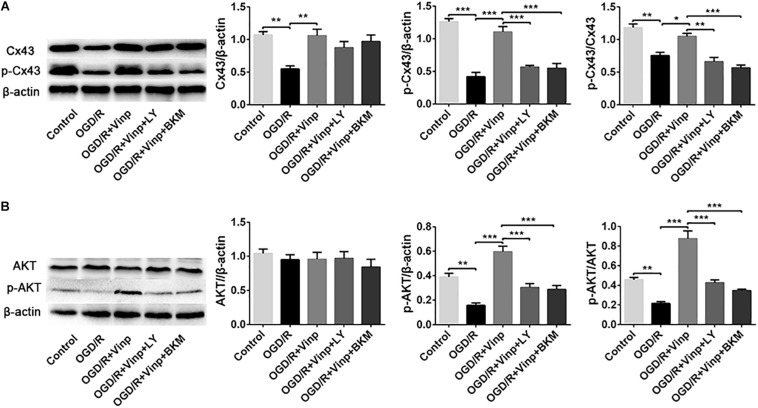
Effects of Vinp on the expression of Cx43, p-Cx43, AKT, and p-AKT in astrocytes after OGD/R. (A) Representative western blot images of Cx43 and p-Cx43, and quantification of Cx43, p-Cx43, and p-Cx43/Cx43. (B) Representative western blot images of AKT and p-AKT, and quantification of AKT, p-AKT and p-AKT/AKT. Data are shown as the mean ± SEM (*n* = 3 in each group). *P < 0.05, **P < 0.01, ***P < 0.001 using one-way ANOVA followed by Tukey’s *post hoc* tests. Vinp: Vinpocetine; LY: LY294002; OGD/R: oxygen-glucose deprivation/reoxygenation; BKM: BKM120.

To further explore whether the abovementioned protective mechanism of Vinp are exerted by targeting the astrocytes, we examined the expression of Cx43, p-Cx43, AKT, and p-AKT *in vitro* astrocyte cultures treated with BKM120 (a specific class I PI3K inhibitor) (Figure 6). The results of Cx43, p-Cx43, AKT, and p-AKT expression, and the ratio of p-Cx43/Cx43 and p-AKT/AKT in each group of *in vitro* cultured astrocytes were consistent with those observed *in vivo*. Moreover, there was no significant difference between the OGD/R + Vinp + LY and OGD/R + Vinp + BKM groups for the abovementioned proteins and their phosphorylation. These results provide more evidence that Vinp protects against cerebral I/R injury by targeting astrocytic Cx43 via the PI3K/AKT pathway.

## Discussion

Ischemic stroke triggers a complex cascade of events, such as excitotoxicity, calcium overload, oxidative stress, inflammation, and apoptosis, which finally leads to dysfunction. For decades, studies on ischemic stroke had mainly focused on neurons. It is a rather recent concept that astrocytes could be a promising therapeutic target for neuroprotection in ischemic stroke (Cekanaviciute and Buckwalter, 2016; Choudhury and Ding, 2016; Liu and Chopp, 2016). One of the mechanisms is that astrocytes could transmit chemical signals or small molecule metabolites through their gap junctions, thereby affecting neuronal survival (Liu and Chopp, 2016).

Vinp was originally invented as a drug for the treatment of diseases caused by cerebrovascular disorders, such as stroke and vascular dementia. Vinp has been shown to be a cyclic nucleotide phosphodiesterase 1 inhibitor (Souness et al., 1989) that also inhibits voltage-dependent Na^+^ channels (Sitges et al., 2005) and IκB kinase (IKK) to exert its anti-inflammatory effects (Jeon et al., 2010). It also exerts significant antioxidant activity by scavenging hydroxyl radicals (Pereira et al., 2000). Although a previous study showed that Vinp can inhibit the inflammatory response caused by cerebral I/R injury and reduce the cerebral infarction volume (Wang et al., 2014), the protective mechanism remains unclear. A study that focused on the role of microglia in the neuroprotection of Vinp proved that microglial TLR4/MyD88/NF-κB is one of the mechanisms by which Vinp protects against cerebral I/R injury (Wu et al., 2017). However, the role of astrocytes in the effect of Vinp against the cascade injury caused by cerebral I/R is unclear.

In this study, we investigated the early protective effects of Vinp *in vivo* and *in vitro* in ischemic stroke models and revealed previously unknown related mechanisms. We found that in cerebral I/R injury rats, Vinp significantly protected against I/R injury by reducing cerebral infarction volume and brain edema. Interestingly, Vinp didn’t significantly reduce the neurological deficit scores, which is contrary to the previous study (Wu et al., 2017). This discrepancy may be a result of the differences in time points and the number of animals used in the two studies. Since the time point of our study *in vivo* was cerebral ischemia for 2 h and reperfusion for 12 h, the degree of neurological deficit may be different at the time point of ischemia for 1 h and reperfusion for 24 h used in the previous study. Furthermore, the number of animals per group was 16 in this study, while it was 7 in the previous study, which may lead to different statistical results. Similarly, findings indicated that Vinp could promote cell survival and reduce cell damage (reduced LDH release) in OGD/R astrocytes. We also explored the neuroprotection of Vinp against the cascade events caused by cerebral I/R injury. Since it is widely considered that the initial step of cerebral I/R is the generation of large amounts of ROS (Dirnagl et al., 1999), we tested the activity of SOD and the content of MDA in the ischemic cortices *in vivo*, and intracellular ROS and NO released into the supernatant *in vitro*. The results revealed that Vinp had an antioxidant effect. It is known that increased ROS may activate astrocytes and microglia, which may produce pro-inflammatory mediators (Chan, 2001; Duris et al., 2018). Blocking the production of pro-inflammatory cytokines would be an important strategy to protect against I/R injury. Thus, we examined the pro-inflammatory cytokines IL-1β and TNF-α by immunofluorescence staining in astrocytes subjected to OGD/R, and the results were consistent with the *in vivo* immunofluorescence double immunostaining with the astrocytic marker GFAP and TNF-α and with the immunoblotting results of pro-inflammatory and anti-inflammatory cytokines. The above *in vivo* and *in vitro* results suggest that Vinp could exert anti-inflammatory effects through astrocytes. Oxidative stress and excessive inflammatory responses could activate pro-apoptotic pathways (Duris et al., 2018), and the activation of caspase-3 is the central part of apoptosis. Thus, we examined the expression of caspase-3 and anti-apoptotic protein Bcl-2 by immunofluorescence staining *in vitro* and immunoblotting *in vivo*. The above results revealed that Vinp attenuated oxidative stress damage, inflammatory responses, and apoptosis both *in vivo* and *in vitro*.

Next, we explored the mechanisms involved in the protection of Vinp. *In vitro* experiments, LY294002 was found to block Vinp’s effects on intracellular ROS and extracellular NO, TNF-α and IL-1β expression, caspase-3 and Bcl-2 expression, astrocytic apoptotic rate. *In vivo* experiments, LY294002 was found to block the effects of Vinp on SOD activity, MDA content, IL-1β, TNF-α, IL-10, BCL-2, and caspase-3 expression. Moreover, *in vivo* immunofluorescence experiments, LY294002 reversed the effect of Vinp on reactive astrocytes, significantly increasing the expression of GFAP and TNF-α and the co-localization of GFAP and TNF-α. Overall, these results indicated that the PI3K/AKT pathway is involved in the anti-oxidative, anti-inflammatory, and anti-apoptotic effects of Vinp against cerebral I/R injury. Additionally, we found that I/R injury resulted in decreased Cx43 expression and enhanced Cx43 dephosphorylation. However, all these changes were inhibited by Vinp, suggesting that Cx43 may play an important role in Vinp’s neuroprotection. More importantly, the inhibition of the PI3K/AKT pathway by LY294002 blocked the above neuroprotective effects of Vinp and reversed the p-Cx43 and p-Cx43/Cx43 changes *in vivo*. We further confirmed the above findings by examining the PI3K/AKT pathway and Cx43 *in vitro* cultured astrocytes with the addition of BKM120 (a specific class I PI3K inhibitor, Burger et al., 2011; Maira et al., 2012). In conclusion, this study showed that Vinp regulates Cx43 in cerebral I/R injury through the PI3K/AKT signaling pathway and provided evidence for its clinical application.

Previous studies have reported that Cx43 is highly phosphorylated under physiological conditions, and ischemia will lead to Cx43 dephosphorylation. Cx43 dephosphorylation is accompanied by the opening of the Cx43 hemichannel, leading to increased influx of several harmful substances and enlargement of the infarct size (Chew et al., 2010). Increased Cx43 expression can reduce neuronal damage after cerebral I/R (Nakase et al., 2003). Our previous studies showed that OGD/R injury can cause Cx43 hemichannel opening and increase in the release of ATP, which could activate the microglia to release numerous inflammatory factors causing neuronal death (Yin et al., 2018). In addition, the inflammatory response of astrocytes increases after ischemic stroke, leading to increased release of extracellular inflammatory factors that affect neuronal survival (Kawabori and Yenari, 2015; Anrather and Iadecola, 2016). Consistent with previous results, we found that cerebral I/R injury downregulated Cx43 and p-Cx43, decreased SOD activity, increased MDA content, decreased the expression of anti-apoptotic protein Bcl-2, and enhanced the expression of apoptotic protein caspase-3 and pro-inflammatory cytokines TNF-α and IL-1β. Effectively, Vinp enhanced Cx43 and p-Cx43 expression and attenuated the aforementioned detrimental effects caused by cerebral I/R, indicating that Vinp likely exerts neuroprotection by targeting Cx43. Studies have shown that Cx43 affects the activation of the inflammasome and the progression of acute kidney injury by regulating the intracellular oxidative status (Huang et al., 2019). Thus, we hypothesized that the protective mechanism of Vinp may be involved in the inhibition of Cx43 internalization and dephosphorylation, accompanied by the closure of the Cx43 hemichannel to reduce intracellular reactive oxygen species, thereby reducing the inflammatory cascade and apoptosis.

Last, we explore the upstream mechanism of Cx43. Cx43 has multiple phosphorylation sites that can be activated by different kinases (including PKA, AKT, and PKC) (Solan and Lampe, 2009), where the C-terminal Ser373 site of the Cx43 can be phosphorylated by AKT (Solan and Lampe, 2014). The PI3K/AKT is an important anti-apoptotic pathway within the cell, and it can induce the formation of IKK by influencing NF-κB and Bcl-2 by phosphorylating GSK-3β, which play a protective role with anti-inflammatory and anti-apoptotic effects (Park et al., 2006; Mullonkal and Toledo-Pereyra, 2007). A previous study showed that PI3K/AKT plays a crucial role in modulating Cx43 expression (Bhattacharjee et al., 2009), conveying mechanical signals to the Cx43 hemichannel and mediating its opening in osteocytes (Batra et al., 2014). Besides, Cx43 has been shown to decrease expression in the heart of *AKT1^–/–^/iAKT2* knockout mice, revealing that AKT plays an important role in maintaining systolic function and Cx43 protein stability (Ock et al., 2018). Inhibition of the PI3K/AKT pathway by LY294002 can reduce Cx43 expression and block the cardioprotective effect of atorvastatin (Bian et al., 2015). Our results indicate that Vinp activates the PI3K/AKT pathway by enhancing the expression of p-AKT (Ser473) to exert anti-oxidative stress and anti-inflammatory effects, thereby exerting anti-apoptotic effects. However, there was no significant change in the expression of AKT, indicating that AKT exerts the above effects through phosphorylation rather than protein expression. Treatment with Vinp and the PI3K/AKT pathway inhibitor LY294002 abolished the upregulation of p-Cx43(Ser373) caused by Vinp after cerebral I/R injury, but not the significant downregulation of Cx43 expression, strongly suggesting that phosphorylation is the manifestation of Cx43 activity. These results were also confirmed *in vitro* using a more than 95% pure primary astrocyte culture. Meanwhile, the expression of AKT and Cx43 induced by the treatment of Vinp and BKM120 (a specific class I PI3K inhibitor,Burger et al., 2011; Maira et al., 2012) was not significantly different than that induced by the treatment of Vinp and LY294002 *in vitro*, which provided further evidence that Vinp targets the PI3K/AKT pathway and regulates the phosphorylation of Cx43. Taken together, our study suggests that the C-terminal Ser373 site of Cx43 can be phosphorylated by AKT activity and plays an important role in the neuroprotection of Vinp.

Our study showed that apoptosis is consistent with changes in proinflammatory factors and oxidative stress, whether in cerebral I/R injury or through the addition of Vinp or LY294002. Previous studies have shown that cerebral I/R injury could induce increased oxidative stress which may induce excessive inflammatory responses that finally activate pro-apoptotic pathways (Dirnagl et al., 1999). Activation of either the Fas, TNF, and TRAIL receptor-mediated extrinsic pathways or direct activation of intrinsic pathways ultimately leads to activation of caspase-3 (Duris et al., 2018). Therefore, we speculate that the anti-apoptotic effect of Vinp may be partly due to its antioxidant and anti-inflammatory effects. Unfortunately, our study could not elucidate the molecular mechanism by which inflammation interacts with apoptosis in the protective effects of Vinp against cerebral I/R. Despite this limitation, this study clearly showed the protective mechanism of Vinp against ischemic stroke.

This study has some other limitations that must be acknowledged. First, we analyzed the early effect of Vinp at cerebral ischemia for 2 h and reperfusion for 12 h based on the previous results of our research group, however, analyzing the effects of Vinp at a longer time point is required. Second, knocking-out the PI3K signaling pathway at the gene level, and not just via PI3K inhibitors, could provide more precise results. Furthermore, further studies are needed to test the effects of Vinp on additional cerebral ischemic models.

## Conclusion

Our study provides new insights into the treatment of ischemic stroke and indicates that Vinp provided neuroprotection against oxidative stress, inflammatory responses, and apoptosis caused by cerebral I/R injury and that this protection may involve astrocytic Cx43 regulation via the PI3K/AKT signaling pathway. Therefore, Vinp could be potentially used to develop a promising drug for the treatment of ischemic stroke.

## Data Availability Statement

All datasets generated for this study are available on request to the corresponding author.

## Ethics Statement

This study was carried out in accordance with the recommendations of China’s Guidelines for Care and Use of Laboratory Animals, the Animals Ethics Committee of Jilin University. The protocol was reviewed and approved by the Animals Ethics Committee of Jilin University.

## Author Contributions

JF, DM, and MZ conceived and designed the experiments. MZ, SH, and LF performed the experiments. MZ, PS, and FW analyzed the data. DN, YZ, and DM contributed the reagents, materials, and analysis tools. MZ, DM, and JF wrote the manuscript.

## Conflict of Interest

The authors declare that the research was conducted in the absence of any commercial or financial relationships that could be construed as a potential conflict of interest.
