# Supplementary figures and images for: Vinpocetine Protects Against Cerebral Ischemia-Reperfusion Injury by Targeting Astrocytic Connexin43 via the PI3K/AKT Signaling Pathway
Source: Front Neurosci. 2020 Apr 2;14:223. doi: 10.3389/fnins.2020.00223 (PMC7142276; doi:10.3389/fnins.2020.00223)

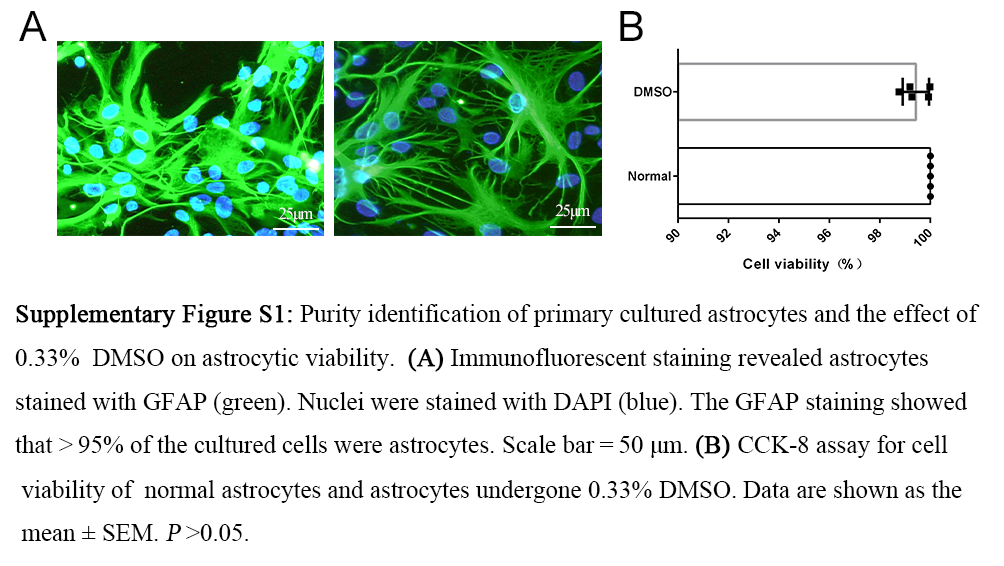

Supplement: Supplementary file 1 [file Image_1.tif]
